# Life-threatening rocuronium-induced anaphylactic shock without cutaneous manifestations successfully reversed with sugammadex: a case report

**DOI:** 10.1186/s40981-020-00402-y

**Published:** 2020-12-07

**Authors:** Yoshiaki Takise, Jungo Kato, Tomohiro Suhara, Takashige Yamada, Takeru Funakoshi, Hayato Takahashi, Masayuki Amagai, Hiroshi Morisaki

**Affiliations:** 1 Department of Anesthesiology, Keio University School of Medicine, 35 Shinanomachi, Shinjuku-ku, Tokyo, 1608582 Japan; 2 Department of Dermatology, Keio University School of Medicine, Tokyo, Japan

**Keywords:** Rocuronium, Sugammadex, Anaphylaxis, Basophil activation test

## Abstract

**Background:**

Recognition of rocuronium-induced anaphylaxis is often challenging, owing to its diverse clinical manifestations. Regarding treatment, several reports have described the efficacy of sugammadex, while conflicting reports have also been published.

**Case:**

A 71-year-old man was scheduled to undergo split-thickness skin grafting surgery on his hip. During the induction of general anesthesia, the patient developed profound circulatory collapse without any cutaneous manifestations, which required 40 min of cardiopulmonary resuscitation. Later, the patient developed circulatory collapse again during the induction of anesthesia for tracheostomy surgery, which apparently coincided with the administration of rocuronium. Rocuronium-induced anaphylactic shock was suspected, and the administration of sugammadex resulted in swift recovery of hemodynamics. The basophil activation test revealed a positive reaction to rocuronium.

**Conclusion:**

The possibility of rocuronium-induced anaphylaxis should be considered when the circulatory collapse coincides with rocuronium administration, even though cutaneous manifestation is absent. Sugammadex can be a treatment option in such atypical cases.

## Background

Anaphylaxis occurring during general anesthesia is a rare, but sometimes life-threatening event that has been reported to occur in up to 1:20,000 cases [1]. Among the causative agents of perioperative anaphylaxis, the most common are neuromuscular-blocking agents (NMBAs), with the highest incidence reported for rocuronium [1]. However, prompt recognition of rocuronium-induced anaphylaxis can be challenging, potentially causing a significant delay in the diagnosis and treatment, as the patients often fail to exhibit the typical cutaneous manifestations of anaphylaxis, such as pruritus and angioedema. Since the introduction of sugammadex as a reversal agent for rocuronium, numerous reports have documented the efficacy of sugammadex for rocuronium-induced anaphylaxis [2–4], although there are conflicting reports concerning its efficacy [5–7].

Herein, we report a patient who developed profound circulatory collapse on two occasions associated with rocuronium-induced anaphylaxis. Informed consent for the publication of this case report was obtained from the patient.

## Case presentation

A 71-year-old man (174 cm, 70 kg) with no known allergies was scheduled to undergo elective split-thickness skin grafting surgery on his hip. The previous skin surgery performed for skin cancer 6 months earlier under spinal anesthesia and inhalational general anesthesia without the use of NMBA had been uneventful. His medical history was also significant for myocardial infarction 15 months earlier that required a percutaneous coronary intervention to the right coronary artery lesion. The latest follow-up coronary angiography revealed 75% stenoses in the left anterior descending artery and in the left circumflex artery. At the time of the current surgery, he was under treatment with aspirin, perindopril, and rosuvastatin. His preoperative screening tests showed no major abnormalities, except for transthoracic echocardiography showing severe hypokinesia of the inferior wall of the left ventricle. The preanesthetic airway assessment revealed no signs of difficult ventilation/intubation (Mallanpati classification I).

**Fig. 1:**
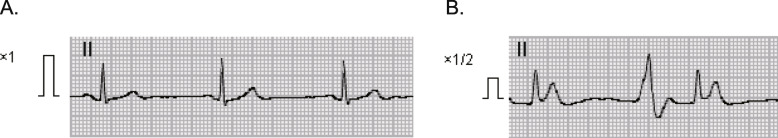
ECG changes during the first episode of circulatory collapse in our patient. **a** Baseline ECG waveform (lead II) showing normal morphology of the QRS complexes and ST-segment. **b** ST-elevation and premature ventricular complexes seen in the early phase of the circulatory collapse

**Fig. 2:**
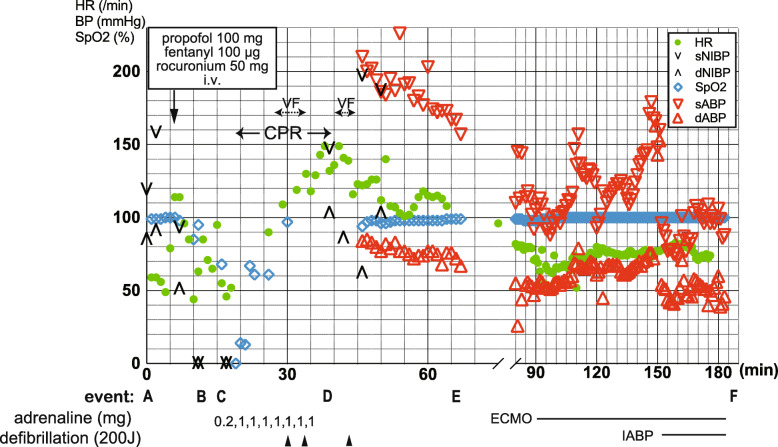
Intractable circulatory collapse following the induction of general anesthesia for skin surgery. **a** Start of anesthesia, (**b**) Oral intubation, (**c**) Emergency call, (**d**) Recovery of spontaneous circulation, (**e**) Transfer to hybrid room, (**f**) Transfer to the intensive care unit. HR, heart rate; BP, blood pressure; SpO_2_, arterial oxygen saturation; s/dNIBP, systolic/diastolic non-invasively measured blood pressure; s/dABP, systolic/diastolic blood pressure measured through the arterial line; VF, ventricular fibrillation; CPR, cardiopulmonary resuscitation; ECMO, extracorporeal membrane oxygenation; IABP, intra-aortic balloon pumping

The standard preanesthetic checkup revealed no significant abnormalities: non-invasive blood pressure, 120/55 mmHg; heart rate, 56 bpm (sinus rhythm); and SpO_2_, 99% on room air. General anesthesia was induced by intravenous administration of 100 mg propofol, 100 μg fentanyl, and 40 mg rocuronium. Immediately after the induction, however, difficulty in mask ventilation was perceived, with a recorded minimal SpO_2_ of 85%. Oral intubation was promptly performed, following which he became profoundly hypotensive, with blood pressure becoming unmeasurable with the non-invasive blood pressure monitor. Meanwhile, the electrocardiogram showed tachycardia (114 bpm) followed by significant bradycardia (44 bpm) with ST-segment elevation (maximal 0.34 μV) (Fig. 1). As his carotid pulse became impalpable, cardiac resuscitation was initiated. Return of spontaneous circulation was eventually obtained following intravenous injection of a total of 7.2 mg adrenaline and two extracorporeal defibrillations for ventricular fibrillation, approximately 40 min after the start of resuscitation. Because the hemodynamics remained unstable, extracorporeal membrane oxygenation (ECMO) and intra-aortic balloon pumping (IABP) were established (Fig. 2). During this entire period of circulatory collapse, the patient did not exhibit any cutaneous manifestations.

Given his past medical history, the circulatory collapse was initially suspected as caused by acute coronary syndrome. However, a coronary angiography performed immediately after the event did not reveal any new positive findings. The skin surgery was canceled, and the patient was transferred to the intensive care unit. He was successfully weaned from ECMO and IABP on post-operative days (POD) 1 and 2, respectively. On the other hand, as his mechanical ventilatory support was expected to be prolonged due to concomitant aspiration pneumonia, a tracheostomy was scheduled on POD 9.

**Fig. 3:**
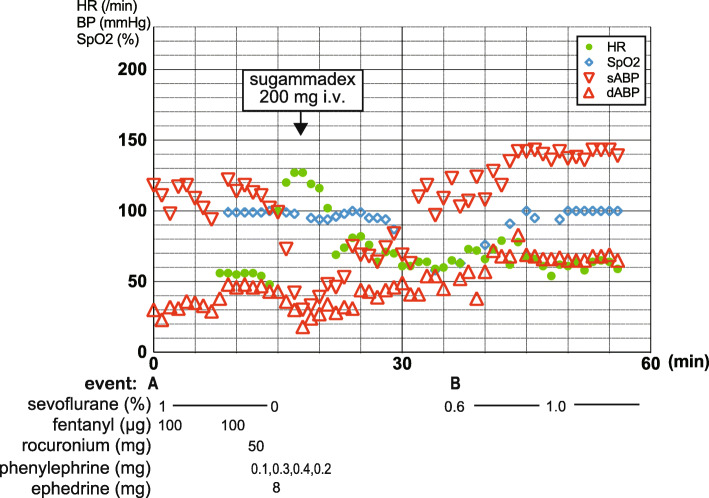
Circulatory collapse following the administration of rocuronium for tracheostomy surgery, which was reversed by sugammadex. The systolic blood pressure decreased to 30 mmHg after the administration of rocuronium 50 mg, which recovered promptly by sugammadex 200 mg. **a** Start of anesthesia, (**b**) Restart of anesthesia. HR, heart rate; BP, blood pressure; SpO_2_, arterial oxygen saturation; s/dABP, systolic/diastolic blood pressure measured through the arterial line

At arrival in the operating room on the scheduled day for tracheostomy, the baseline vital parameters were as follows: arterial blood pressure, 122/48 mmHg; HR, 56 bpm (sinus rhythm); and SpO_2_, 99% under an FiO_2_ of 40%. General anesthesia was induced by the administration of sevoflurane via the endotracheal tube. Then, following the administration of 50 mg rocuronium, the invasive arterial blood pressure monitor showed a sudden precipitous decrease up to 30/18 mmHg, which proved refractory to repeated intravenous injections of ephedrine (up to a total of 8 mg) and phenylephrine (up to a total of 0.8 mg). Although the patient still did not exhibit any cutaneous manifestations, rocuronium-induced anaphylaxis was suspected, as the onset of circulatory collapse appeared to be associated with rocuronium administration on both occasions. Consequently, 6 min after the rocuronium administration, 200 mg sugammadex was administered by intravenous injection. Apparently, coincidentally with the intravenous administration of sugammadex, the patient’s hemodynamics began to show sudden dramatic improvement, to the extent that further vasopressor administration was no longer needed (Fig. 3). The tracheostomy was performed successfully, as planned.

Laboratory examination revealed a marked elevation of the serum tryptase level to 7.7 μg/dL at 14 h after the tracheostomy. In addition, the basophil activation test (BAT) revealed a strong positive reaction to rocuronium, indicating that the repetitive episodes of circulatory collapses were attributable to rocuronium-induced anaphylaxis. Skin testing was avoided due to the potential risk of a fatal allergic reaction. The post-operative course was uneventful, and the patient was discharged home on POD 40 with no discernible sequelae.

## Discussion

In the current case, we encountered abrupt life-threatening circulatory collapse immediately following the induction of general anesthesia on two occasions, which were eventually found to be attributable to rocuronium-induced anaphylaxis.

The absence of the typical cutaneous manifestations of anaphylaxis significantly delayed our recognition of anaphylaxis. While cutaneous manifestations, such as pruritus and angioedema, are commonly observed in patients with anaphylaxis, the current guidelines warn that approximately 10% of patients with anaphylaxis may not exhibit the typical cutaneous symptoms/signs, especially when the peripheral circulation is severely compromised [8]. Moreover, a recent survey demonstrated that only 20% of patients with rocuronium-induced anaphylaxis exhibited skin manifestations [1]. Therefore, it is crucial for the anesthesiologist to bear a high index of suspicion for rocuronium-induced anaphylaxis, in a patient developing circulatory collapse coincidentally with rocuronium administration, even in the apparent absence of cutaneous manifestations.

Classically, IgE/Fcε receptor-mediated activation of mast cells/basophils has been proposed to be the main mechanism underlying rocuronium-induced anaphylaxis [9]. The quaternary ammonium structure of rocuronium is considered to be the major epitope in the IgE-mediated reactions [10]. Indeed, the elevated serum tryptase level and the positive result of the BAT support the involvement of mast cell/basophil activation in the present case. On the other hand, IgE-independent mechanisms, including direct activation of mast cells/basophils via Mas-related G-protein-coupled receptor X2 (MRGPRX2) and rocuronium-specific IgG-mediated neutrophil activation, have also been implicated [11]. Given the lack of the typical cutaneous manifestations, such non-canonical pathways were probably involved in the current case.

Another possible mechanism for profound circulatory collapse could be anaphylaxis-related coronary arterial vasospasm, which is designated as Kounis syndrome [12]. A previous report described the occurrence of rocuronium-induced Kounis syndrome [13]. Despite the negative findings of coronary angiography, the development of Kounis syndrome could also have contributed to the profound circulatory collapse in the present case, given the previous history of coronary artery disease and the ST-segment elevation observed during the circulatory collapse (Fig. 1).

In sharp contrast to the first episode, the swift recovery of the hemodynamics following sugammadex administration after the second episode of circulatory collapse strongly suggests the therapeutic potential of sugammadex for rocuronium-induced anaphylaxis. Numerous reports have documented successful reversal of rocuronium-induced anaphylaxis following sugammadex administration [3, 4]. The dramatic response in our case indicates that sugammadex may also be effective in cases of atypical anaphylaxis that fail to show cutaneous manifestations. An in vitro study suggests that pre-incubation with sugammadex is effective for preventing the rocuronium-induced anaphylactic processes [5]. Conversely, other reports have suggested that sugammadex administration failed to reverse already-activated basophils or cutaneous manifestations in rocuronium-allergic patients [6, 7]. Considering the findings collectively, we also doubt that sugammadex can halt the reaction of already-activated mast cells/basophils. Rather, when it is given shortly after the rocuronium administration, the encapsulation of unbound rocuronium may have been responsible for preventing further activation of the anaphylactic cascade, thereby making vasopressor treatment more effective. In addition, its effects on alternative mechanisms, such as IgG-mediated pathway or MRGPRX2-mediated direct activation, remain entirely unknown.

Moreover, the dose of sugammadex for rocuronium-induced anaphylaxis remains open to question. The effective doses on rocuronium-induced anaphylaxis range from 4 to 18 mg/kg in previous case reports [14]. Assuming that sugammadex (2178 Da) encapsulates rocuronium (610 Da) at a molar ratio of 1:1, theoretically, 3.57 mg of sugammadex would be needed per milligram of rocuronium. In the present case, the administration of 200 mg sugammadex 6 min after the administration of 50 mg of rocuronium was sufficient to halt the rocuronium-induced circulatory collapse. Because of the linear, dose-dependent pharmacokinetics of sugammadex [15], we believe a higher dose (e.g., 16 mg/kg) might have been more efficient, with faster and more definite action. In addition, the timing of sugammadex administration may also be important, as profound circulatory failure may delay the distribution of sugammadex, thereby slowing its therapeutic effect.

Taken together, our case illustrates major challenges associated with rocuronium-induced anaphylaxis developing during induction of general anesthesia, particularly when cutaneous manifestations are absent. Our experience suggests that sugammadex can be an effective treatment option for rocuronium-induced anaphylaxis in selected cases. Nevertheless, further accumulation of evidence is necessary to determine the patient selection and the optimal dose and timing of administration of sugammadex for rocuronium-induced anaphylaxis.

## Data Availability

The data in this case report are available from the corresponding author on reasonable requests.

## Abbreviations

NMBA: Neuromuscular-blocking agent
ECMO: Extracorporeal membrane oxygenation
IABP: Intra-aortic balloon pumping
POD: Post-operative day
BAT: Basophil activation test
MRGPRX2: Mas-related G-protein-coupled receptor X2
